# Not All Are Lost: Interrupted Laboratory Monitoring, Early Death, and Loss to Follow-Up (LTFU) in a Large South African Treatment Program

**DOI:** 10.1371/journal.pone.0032993

**Published:** 2012-03-12

**Authors:** Aima A. Ahonkhai, Farzad Noubary, Alison Munro, Ruth Stark, Marisa Wilke, Kenneth A. Freedberg, Robin Wood, Elena Losina

**Affiliations:** 1 Division of Infectious Disease, Massachusetts General Hospital, Boston, Massachusetts, United States of America; 2 Division of General Medicine, Massachusetts General Hospital, Boston, Massachusetts, United States of America; 3 Medical Practice Education Center, Massachusetts General Hospital, Boston, Massachusetts, United States of America; 4 Southern African Catholic Bishops' Conference, Pretoria, Gauteng, South Africa; 5 Catholic Relief Services South Africa, Johannesburg, Gauteng, South Africa; 6 Department of Health Policy and Management, Harvard School of Public Health, Boston, Massachusetts, United States of America; 7 Harvard University Center for AIDS Research, Boston, Massachusetts, United States of America; 8 Department of Orthopedic Surgery, Brigham and Women's Hospital, Boston, Massachusetts, United States of America; 9 Desmond Tutu HIV Centre, Institute for Infectious Disease and Molecular Medicine, University of Cape Town, Cape Town, Western Cape, South Africa; 10 Department of Medicine, University of Cape Town, Cape Town, Western Cape, South Africa; 11 Department of Biostatistics, Boston University School of Public Health, Boston, Massachusetts, United States of America; 12 Department of Epidemiology, Boston University School of Public Health, Boston, Massachusetts, United States of America; University of Cape Town, South Africa

## Abstract

**Background:**

Many HIV treatment programs in resource-limited settings are plagued by high rates of loss to follow-up (LTFU). Most studies have not distinguished between those who briefly interrupt, but return to care, and those more chronically lost to follow-up.

**Methods:**

We conducted a retrospective cohort study of 11,397 adults initiating antiretroviral therapy (ART) in 71 Southern African Catholic Bishops Conference/Catholic Relief Services HIV treatment clinics between January 2004 and December 2008. We distinguished among patients with early death, within the first 7 months on ART; patients with interruptions in laboratory monitoring (ILM), defined as missing visits in the first 7 months on ART, but returning to care by 12 months; and those LTFU, defined as missing all follow-up visits in the first 12 months on ART. We used multilevel logistic regression models to determine patient and clinic-level characteristics associated with these outcomes.

**Results:**

In the first year on ART, 60% of patients remained in care, 30% missed laboratory visits, and 10% suffered early death. Of the 3,194 patients who missed laboratory visits, 40% had ILM, resuming care by 12 months. After 12 months on ART, patients with ILM had a 30% increase in detectable viremia compared to those who remained in care. Risk of LTFU decreased with increasing enrollment year, and was lowest for patients who enrolled in 2008 compared to 2004 [OR 0.49, 95%CI 0.39–0.62].

**Conclusions:**

In a large community-based cohort in South Africa, nearly 30% of patients miss follow-up visits for CD4 monitoring in the first year after starting ART. Of those, 40% have ILM but return to clinic with worse virologic outcomes than those who remain in care. The risk of chronic LTFU decreased with enrollment year. As ART availability increases, interruptions in care may become more common, and should be accounted for in addressing program LTFU.

## Introduction

In 2009, South Africa was home to the greatest number of people living with HIV in the world – 5.7 million [1]. In response to this epidemic, South Africa initiated the largest HIV treatment program in the world, placing 1 million people on antiretroviral therapy (ART) by the end of 2009 [1].

Despite tremendous advances in the scope and reach of HIV care and treatment in South Africa and other resource-limited countries, high rates of loss to follow-up (LTFU) have been reported in systematic reviews of the literature, approaching 70% before ART initiation and approximately 25% one to three years after ART initiation [2], [3]. Up to 50% of patients who are lost to follow-up in some programs are later found to be dead, with many deaths occurring in the first 6 months after ART initiation [4]. LTFU has, therefore, been increasingly recognized as an important programmatic challenge, and has been regarded as a measure of program effectiveness [4]–[6]. However, because of the inherent challenges in ascertaining “true” patient outcomes in resource-limited settings, LTFU populations from any given HIV treatment program may unknowingly include heterogeneous groups of patients with distinct outcomes. Such outcomes include patient deaths, undocumented transfers of care to other sites, and transient interruptions in care, in addition to actual loss of contact with patients [7].

Routine patient monitoring is accepted as a cornerstone of HIV disease management and program evaluation [8]. While the implications of chronic LTFU and unstructured ART interruption have received substantial attention, and include increased risk of treatment failure and death, the implications of brief interruptions in care, which may include any combination of non-compliance with clinical visits, laboratory assessments, or ART are less certain [9]. Two randomized trials conducted in resource-limited settings suggest that when compared to clinical monitoring alone routine laboratory monitoring plays an important role in the early recognition of treatment failure and prevention of progression to AIDS and death [10], [11]. Patients with brief interruptions in chronic care, including laboratory monitoring, may also be at increased risk for poor outcomes. Definitions of LTFU, however, are not standardized across studies with respect to time since last visit, and most studies do not account for such patients who interrupt, but then return to care [12].

Our objective was to study patients newly initiated on ART with suboptimal adherence to HIV care – either with brief interruptions in care or chronic LTFU – in addition to patients with early mortality. We investigated patient and clinic level factors associated with these patient groups, and virologic response among those in care and those with interrupted care.

## Methods

### Ethics Statement

IRB approval was obtained from the Massachusetts General Hospital and from the University of Cape Town.

### Setting

The Catholic Relief Services and Southern African Catholic Bishops Conference (CRS/SACBC) are faith-based, non-governmental organizations that have been providing care to HIV-infected individuals in South Africa since 2003. The PEPFAR-funded HIV treatment program commenced largely in concert with South Africa's 2004 national ART roll-out. It was initially launched in 20 facilities, most of which were pre-existing home-based care programs run by the Catholic Church. The program is now comprised of 71 sites (14 central and 57 satellite clinics), which have provided care to over 70,000 patients since 2004 [13]. These treatment sites span 8 of the 9 South African provinces. They are situated in rural, urban, peri-urban, squatter, as well as mining communities, and provide services to children, adults, and pregnant women. HIV services are provided free of charge in hospitals, primary health clinics, primary HIV clinics, and residential facilities. Clinics are staffed by teams typically including at least one part-time doctor and two nurses, in addition to counselors and adherence monitors. Treatment protocols closely follow South African National Department of Health and World Health Organization guidelines [14]. In addition to ART services, all programs provide voluntary testing and counseling, while some also provide inpatient “hospice” treatment, residential homes for orphans and vulnerable children, educational programs, drug and alcohol rehabilitation services, and other community development initiatives.

### Study Sample

The study population is comprised of adults (≥15 years) who enrolled in the CRS/SACBC treatment programs between January 2004 and December 2008. Patients included in the analysis were those who were eligible for ART at clinic enrollment (baseline CD4 count <200/uL or WHO stage III or IV disease), subsequently initiated ART, and had at least 400 days of potential follow-up time on ART before the end of the study. We included a minimum follow-up time in order to have an observation period sufficient to capture brief interruptions in care.

### Data Elements

After clinic enrollment, patients received baseline clinical assessment and CD4 count testing. Those with CD4 counts ≤200/uL or WHO stage III or IV disease, were started on a standard regimen of stavudine, lamivudine, and efavirenz (unless contraindicated) after completing ART literacy training. Upon ART initiation, patients received monthly adherence assessments, 3 to 6 monthly clinical follow-up, and 6 monthly laboratory follow-up with CD4 testing, and, in some cases, HIV RNA monitoring. Only those clinic visits associated with routine laboratory monitoring were entered into the database and available for analysis. An adherence counselor typically contacted patients who defaulted from care either by phone, home-visit, or both. The details of this practice varied by clinic. A minimum set of demographic and clinical variables was collected at each clinic site and entered into a standardized program database.

### Definitions

A patient visit was defined as a follow-up appointment associated with CD4 monitoring. Since first published in 2004, South African national HIV treatment guidelines have recommended 3-monthly clinical follow-up and 6-monthly CD4 monitoring [15]. Patients were categorized into one of four mutually exclusive groups reflecting concordance with these guidelines. Those *in care* were guideline-concordant. They were alive and had at least one visit between 30 and 210 days after ART initiation. Patients with *interrupted laboratory monitoring (ILM)* had no follow-up visits between 30 and 210 days after ART initiation, but returned to clinic for laboratory monitoring by 400 days. *LTFU* was defined as having no follow-up visits between 30 and 400 days after ART initiation. Patients with *early death* had a documented death within the first 210 days after starting ART. Patient deaths were ascertained passively. Clinics were notified of patient deaths by home-based caregivers, family members, or by residential facilities associated with the clinics. Patients with documented transfers to another facility were assumed to be in care for this analysis.

### Statistical Analysis

Bivariate and multivariate, multilevel multinomial logistic regression modeling was used to assess the relationship of patient-level factors (age, gender, baseline CD4 count – the first CD4 count after clinic enrollment, year of enrollment) and clinic-level factors (urban, peri-urban, or rural, number of patients on ART, time to ART initiation) on the odds of ILM, LTFU, and dying within 7 months of ART initiation, compared to remaining in care. Multilevel modeling was required to account for patient clustering within satellite clinics, which were clustered within primary clinics [16]. Multinomial modeling was required to compare multiple outcomes (ILM, LTFU, early death) with the reference group (in care) [16]. Logistic regression was favored over time-to-event analysis to allow us to characterize patient experiences in the first year on ART, and to assess differences in outcomes across patient enrollment year. Covariates with associations at p≤0.10 in any of the univariate models were included in the final multivariate models. Four separate Cochran-Armitage tests for trend (one for each of the outcomes against all other outcomes combined) were conducted to assess changes in the distribution of outcomes with patient enrollment year [17], [18]. The Bonferroni correction was used to account for multiple comparisons, and, as a result, these tests were assessed at the 0.05/4 = 0.0125 significance level [19]. A test of proportions was used to compare the proportion of patients with detectable viral loads (HIV RNA >400 copies/uL) at 12 months of follow-up on ART of patients in care to those with ILM. By definition, LTFU patients did not have a follow-up visit at 12 months, so a comparison with this group was not performed. Data were analyzed using SAS version 9.2, SAS Institute Inc. 2008.

## Results

### Cohort Description

Between 2004 and 2008, there were 22,888 ART-naive adults enrolled in the CRS/SACBC HIV treatment programs with advanced HIV disease (CD4 count ≤200/uL or WHO stage III or IV), for whom data were available. The analysis focused on 11,397 persons who initiated ART and had at least 400 days of potential observation time after ART initiation and before the end of the study. Follow-up data were restricted to the first 400 days after ART initiation for all patients. Thirty-eight percent of patients were enrolled in urban programs, 44% in peri-urban programs, and 18% in rural programs.

The number of ART-naïve, ART-eligible patients enrolled in central clinics over the study period ranged from 372 at the smallest site to 5153 at the largest. On average, 65% of ART-eligible patients were initiated on ART.

### Baseline Patient Characteristics

The majority of patients were female (67%). Median age was 35 years, and median baseline CD4 count at enrollment was 101/uL [IQR: 43/uL,160/uL] (Table 1). CD4 count was 108/uL [IQR: 47/uL, 166/uL] among patients who interrupted laboratory monitoring and 48/uL [IQR: 15/uL, 106/uL] among patients who died in the first 7 months after ART initiation. The median time from clinic enrollment to ART initiation was 34 days [IQR: 17 days, 65 days]. This duration ranged from a median of 28 days [IQR: 14 days, 53 days] for patients with early death to 35 days [IQR: 20 days, 70 days] for patients in care.

**Table 1:** Summary of baseline patient and clinic characteristics in a cohort study of LTFU in a large South African ART program.

Characteristics		Overall (n = 11,397)	In Care (n = 7,215)	Interrupted LaboratoryMonitoring (n = 1,236)	LTFU (n = 1,958)	Early Death (n = 988)
Age median years [IQR]		35 [30, 42]	35 [30, 43]	35 [30, 41]	34 [29, 41]	36 [30, 43]
Sex	% Female	66.5	67.8	63.9	65.6	62.3
	% Male	33.5	32.2	36.1	34.4	37.7
Enrollment HIV RNA median log_10_ copies/mL		4.93	4.91	4.91	4.89	5.13
Enrollment CD4 count median/uL [IQR]		101 [43, 160]	107 [49,164]	108 [47, 166]	103 [44, 161]	48 [15, 106]
Enrollment Year	% 2004	6.6	6.4	5.3	7.6	8.2
	% 2005	19.9	19.2	19.9	19.6	25.2
	% 2006	21.0	20.8	19.3	22.6	21.5
	% 2007	25.8	26.3	20.6	28.6	23.2
	% 2008	26.7	27.3	34.9	21.6	21.9
Geographic description	% Urban	38.2	36.9	44.1	58.3	21.6
	% Peri-Urban	43.7	47.1	32.2	22.5	71.1
	% Rural	18.1	16.0	23.7	19.2	7.3
Number of patients on ART	% ≤100	30.5	8.5	14.2	18.6	7.4
	% 101–500	24.1	26.7	16.8	13.5	34.4
	% 501–1000	11.8	25.4	25.1	14.4	33.4
	% >1000	33.6	39.4	43.9	53.5	24.8
Days to ART initiation median [IQR]		34 [17, 65]	35 [20, 70]	34 [14, 64]	29 [14, 61]	28 [14, 53]

LTFU: Loss to Follow Up, ART: Antiretroviral Therapy, IQR; Interquartile Range.

### Patient Outcomes

**Figure 1:**
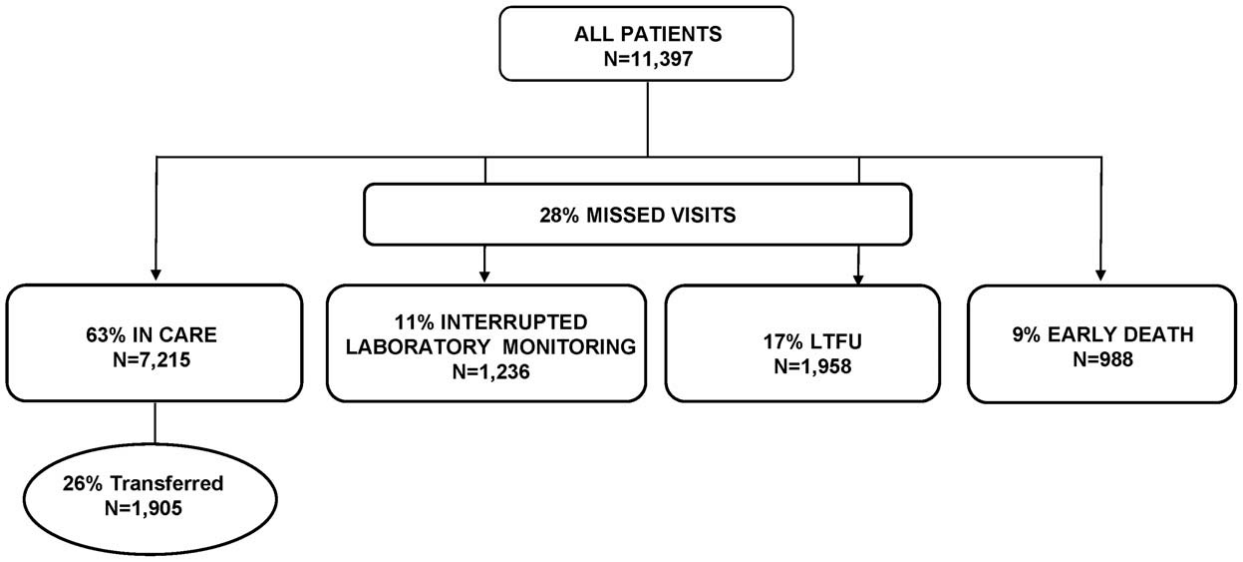
Summary of patient outcomes in a cohort study of LTFU in a large South African ART program.

At the end of the study period, 63% of patients remained consistently in care (n = 7,215), 28% (n = 3,194) missed visits, and 9% (n = 988) had early death. Of the patients in care, 26% (n = 1,905) had documented transfers to another facility after a median of 399 days on ART. Of the 3,194 patients who missed visits 1,236 (11% of cohort) had ILM, and 1,958 (17% of cohort) were LTFU (Figure 1). Across all central clinics, the proportion of patients classified as LTFU ranged from 1% to 35%; those with ILM ranged from <1% to 26%, and early deaths ranged from <1% to 22%. Patients who interrupted laboratory monitoring had an increase in median CD4 count from 108/uL at baseline to 257 cells/uL when monitoring was resumed. Of note, 416 patients (4% of cohort) had late deaths, after the first 7 months on ART. These deaths occurred a median of 436 days after ART initiation. Given that outcomes were ascertained after 400 days of follow-up, these patients were assigned to the outcome (in care, ILM, or LTFU) that applied at the time of study censorship.

### Trends in Outcomes Over Time

**Figure 2:**
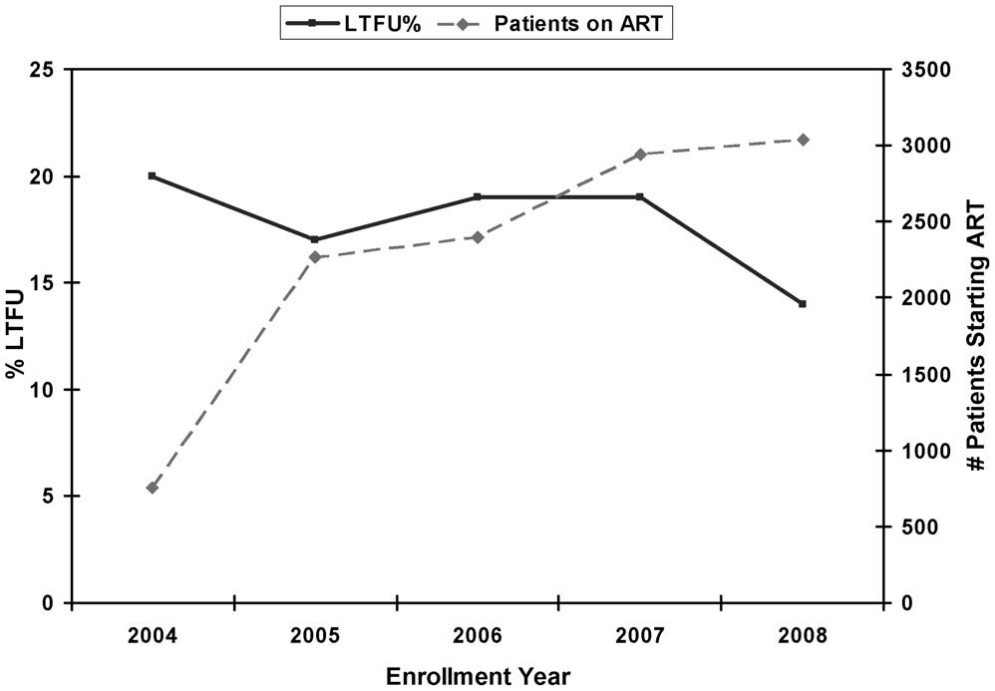
Trends in LTFU and program size in the Catholic Relief Services/Southern African Catholic Bishops Conference HIV treatment program, 2004–2008.

The number of patients that initiated ART in the CRS/SACBC clinics increased with each calendar year, from 753 in 2004 to 3,042 in 2008 (Figure 2). Over this period, the proportion of patients who remained in care increased from 61% in 2004 to 65% in 2008 (p-value for Cochran-Armitage test for trend = 0.0190; not significant at the 0.0125 level). There was also an overall increase in the proportion of patients who had ILM, from 9% to 14% (p-value = 0.1128), and significant decreases in both LTFU, from 20% to 14% (p-value = 0.0016), and in early deaths, from 11% to 7% (p-value = <0.0001) (Figure 2). During the same time, there was a small decline in the proportion of patients presenting to clinic with advanced disease. In 2004, 54% of patients presented with a baseline CD4 count ≤100/uL, compared to 49% in 2008.

In multivariate analysis, later year of enrollment was associated with decreased risk of LTFU. Compared to patients enrolled in 2004, patients enrolled in 2008 had a 51% reduction in the risk of LTFU [OR 0.49; 95%CI 0.39–0.62]. We did not find similar trends related to ILM or early death.

### Detectable Viral Load at 12 Months

Of all patients who remained in care, 71% had a viral load obtained a median of 376 days after ART initiation; 13% had detectable viremia (HIV RNA >400 copies/uL). Among patients with ILM, 64% had a viral load obtained a median of 393 days after ART initiation; 17% had detectable viral loads. There was a 30% increase in detectable viremia at 12 months among patients with ILM compared to patients in care (p = 0.0112).

### Factors Associated with Interruped Laboratory Monitoring, LTFU, or Death

In multivariate analysis, female sex was associated with a decreased odds of ILM [OR 0.80; 95%CI 0.70–0.92], LTFU [OR 0.82; 95%CI 0.73–0.91], and early death [OR 0.86; 95%CI 0.75–0.99] compared to male sex. Aside from female sex, we did not find any other patient-level factors associated with ILM (Table 2).

**Table 2:** Factors associated with interrupted laboratory monitoring, LTFU, and early death in multivariate analysis*.

		Interrupted laboratory monitoring		LTFU		Early Death	
		OR	*p*	OR	*p*	OR	*p*
Age (years)	>35	1.00		1.00		1.00	
	≤35	1.03 [0.91–1.17]	0.6356	1.07 [0.97–1.19]	0.1940	1.01 [0.88–1.17]	0.8507
Sex	Male	1.00		1.00		1.00	
	Female	**0.80 [0.70–0.92]**	**0.0012**	**0.82 [0.73–0.91]**	**0.0003**	**0.86 [0.75–0.99]**	**0.0453**
Enrollment CD4 (cells/uL)	>200	1.00		1.00		1.00	
	101–200	0.93 [0.74–1.17]	0.5065	1.01 [0.83–1.23]	0.9009	**2.16 [1.38–3.38]**	**0.0007**
	50–100	0.84 [0.66–1.08]	0.1748	1.03 [0.83–1.27]	0.8060	**3.64 [2.32–5.72]**	**<0.0001**
	<50	1.00 [0.79–1.27]	0.9886	1.21 [0.99–1.49]	0.0698	**6.43 [4.13–9.99]**	**<0.0001**
Enrollment Year	2004	1.00		1.00		1.00	
	2005	1.21 [0.90–1.63]	0.1989	0.96 [0.77–1.19]	0.6963	1.07 [0.79–1.45]	0.6609
	2006	0.90 [0.66–1.21]	0.4689	**0.80 [0.64–0.99]**	**0.0468**	1.00 [0.73–1.36]	0.9816
	2007	0.76 [0.56–1.02]	0.0681	**0.66 [0.53–0.83]**	**0.0003**	1.02 [0.75–1.39]	0.8801
	2008	1.25 [0.93–1.67]	0.1362	**0.49 [0.39–0.62]**	**<0.0001**	0.90 [0.66–1.22]	0.4962
Geographic Description	Urban	1.00		1.00		1.00	
	Peri-Urban	0.69 [0.28–1.70]	0.4187	**0.25 [0.10–0.59]**	**0.0017**	1.71 [0.59–4.90]	0.3210
	Rural	1.23 [0.40–3.77]	0.7153	0.45 [0.17–1.19]	0.1056	0.71 [0.20–2.57]	0.6014
Number of Patients on ART	≤100	1.00		1.00		1.00	
	101–500	**0.63 [0.42–0.93]**	**0.0191**	**0.53 [0.29–0.97]**	**0.0396**	0.72 [0.35–1.50]	0.3866
	501–1000	0.82 [0.43–1.56]	0.5415	0.58 [0.23–1.48]	0.2533	1.53 [0.55–4.29]	0.4200
	>1000	**0.52 [0.32–0.85]**	**0.0083**	0.74 [0.31–1.74]	0.4839	1.28 [0.51–3.24]	0.5980
Days to ART Initiation	0–30	1.00		1.00		1.00	
	>30	0.90 [0.79–1.03]	0.1247	0.92 [0.83–1.03]	0.1294	**0.78 [0.67–0.90]**	**0.0006**

LTFU: Loss to Follow Up, ART: Antiretroviral Therapy, OR: Odds Ratio,

*all models adjusted for all variables listed in table.

Baseline CD4 count was a predictor of early death, but not LTFU. In univariate analysis, patients with baseline CD4 <50/uL had an increased risk of LTFU [OR 1.3; 95%CI 1.06–1.61] compared to those with baseline CD4 >200/uL, but this relationship did not persist in the multivariate model [OR 1.21; 95%CI 0.99–1.49]. In multivariate analysis, the risk of early death increased with decreasing baseline CD4 count [OR 6.43; 95%CI 4.13–9.99], for patients with baseline CD4 <50/uL compared to those with baseline CD4 >200/uL.

Among all factors assessed, we did not find any clinic characteristics associated with higher rates of LTFU. Longer time to ART initiation was associated with a decreased risk of early death [OR 0.78; 95%CI 0.67–0.90], and there was a trend suggesting decreased risk of ILM among patients attending larger programs (>100 patients on ART), (Table 2).

When patients with *late* deaths (after 7 months on ART) were analyzed in a separate multinomial, multivariate model comparing ILM, LTFU, early death, and late death with the reference group (in care), we saw no change in the factors associated with ILM, LTFU, and early death. Enrollment year after 2005 was associated with a decreased odds of late death; patients enrolled in 2006, 2007, and 2008 had a 49% [95%CI 28–64], 70% [95%CI 57–79], and 76% [95%CI 65–83] reduction in the odds of late death compared to those in care. Additionally, patients with a baseline CD4 count ≤100/uL had an increased risk of late death with OR 1.66 [95%CI 1.09–2.54] for patients with baseline CD4 50–100/uL, and OR 1.75 [95%CI 1.14–2.62] for patients with baseline CD4 count <50/uL.

## Discussion

In an analysis of over 11,000 patients newly-initiated on ART between 2004 and 2008 across South Africa, 63% of patients remained stably in care in the first year after ART initiation. Nine percent of patients suffered early death within the first seven months after ART initiation. These patients presented with very advanced disease, with a median CD4 count of 48/uL. Missed visits were seen in 28% of patients. Eleven percent of patients had ILM but returned to care by 400 days, and there was a trend towards increase over time. Importantly, patients with ILM would often have been categorized as LTFU if the analysis did not account for those who resumed laboratory monitoring after an initial absence from care, and would have increased the “LTFU” rate in this analysis from 17% to 28%. In addition, the majority of patients with interruptions had an increase in CD4 count and had suppressed HIV viral load upon return to care, suggesting that they had continued ART during the period of interrupted monitoring. Despite this, patients with ILM had a lower rate of virologic suppression at 12 months (83%) compared to patients who remained in care (87%). Seventeen percent of patients were lost to follow up at one year, but the odds of LTFU decreased significantly with more recent enrollment year. As in several other studies, women had a decreased risk of LTFU, ILM and death [20], [21]. While low baseline CD4 count predicted early death, we did not identify any other baseline patient-level factors that would distinguish patients with ILM from those ultimately lost to follow-up.

With the critical goal of retaining patients in HIV care over time, LTFU has been recognized as an important programmatic metric. Patient tracing studies confirm that many patients identified as lost to follow up have died (7%–40%), transferred care (30%), or voluntarily withdrawn from care [6], [22]–[24]. Each of these types of patients has different outcomes, challenging the notion that all LTFU reflects a poor outcome. In the current study, which excluded known early deaths and patients with ILM, we did not find low baseline CD4 count to be associated with higher rates of LTFU. The same has been seen in other studies that exclude known deaths, particularly early deaths, from the definition of LTFU [25], [26].

Despite a growing body of literature on LTFU in HIV treatment programs, patients that interrupt, but subsequently return to care, have received relatively little attention. While failure to attend clinic appointments is common in the management of chronic illness, there are few data about patients with ILM in resource-limited settings [27]–[29]. In the current study, of all patients who missed visits in the first 7 months on ART, nearly 40% returned to clinic for monitoring and care by one year. Another South African study found that of the one-third of patients who defaulted from care, another one-third resumed ART after a median of 228 days [21]. In that study, patients lost the immunologic benefit of ART, with CD4 counts at return comparable to the pre-ART CD4 counts [21]. In contrast, most patients in our cohort with ILM had an increase in CD4 count and decrease in HIV RNA upon resumption of care, suggesting that the vast majority remained on ART. Despite this increase in CD4, patients with ILM had lower rates of virologic suppression at 12 months compared to those who remained in care. These findings underscore the importance of compliance with a package of care services that include clinical and laboratory assessment. Indeed, two randomized controlled trials with 3 to 5 years of patient follow-up found that routine laboratory monitoring (with CD4 and or viral load testing) was associated with improved health and survival when compared to clinical monitoring alone in resource-limited settings [10], [11]. The authors of the DART study, which compared laboratory monitoring to clinically driven monitoring in Zimbabwe, reported that 59 patients would need to be monitored for 1 year to avoid one new WHO stage 4 event or death [11].

It is possible that patients with ILM sought care or obtained ART from other facilities. This phenomenon may be especially relevant for highly mobile populations and in communities where expansion in HIV treatment facilities provides patients with the option to choose clinics that best meet their needs. It is also possible that some patients came to clinic visits only, but did not complete laboratory follow-up, or that incomplete record-keeping led some patients to be misclassified, despite complying with routine laboratory monitoring. One study from South Africa found that among patients classified as lost to follow-up in an HIV treatment program, 21% were still in care – half were incorrectly identified as lost, and the other half had interrupted care but returned [24]. These findings underscore the prevalence of missed visits in HIV programs in resource-limited settings, as well as the importance of data quality on the assessment of patient outcomes.

While many studies have investigated the association between patient characteristics and LTFU, fewer have measured the association between program characteristics and this risk. One large meta-analysis of 33 patient cohorts in 13 Sub-Saharan African countries found no association between cohort size or year of program initiation and LTFU. But the ART-LINC study of 23 treatment programs in low and middle-income countries found that larger programs had higher rates of LTFU and were less likely to trace patients who were lost [30]. In the current study, only patient-level factors predicted LTFU. We did not find a relationship between program size, geographic description, or time to ART initiation and LTFU.

Definitions of LTFU may also affect the assessment of temporal trends of important patient outcomes. Our analysis showed a decrease in LTFU and patient deaths, but an increase in ILM, during the period of study. This contrasts with a study from the IeDEA-SA cohort, which similarly showed a decline in patient mortality, but an increase in LTFU, from 1% to 13%, between 2002 and 2007 [21]. In this study of eight public sector programs, the authors suggested that rapid program growth over this period, and subsequent strain on monitoring systems, might explain the increase in LTFU [21]. While we also found an increase in enrollment by nearly 300% from 2004 to 2008, there are several potential reasons for these conflicting results. First, we excluded ILM from our definition of LTFU. Increases in ILM over time may contribute to increasing rates of “LTFU,” if these patients are included in the definition of LTFU. Second, over the course of our study period, the CRS/SACBC programs witnessed substantial improvements in their patient record system and developed a more intensive home-based care program better equipped to identify early patient defaulters, complete home visits, and encourage patients to return to care. In fact, the home-based care networks in many of these communities preceded HIV treatment services, and the longstanding relationships between the church and surrounding communities may foster different dynamics than those seen in public sector programs.

This study has several limitations. First, we did not have systematic ascertainment of patient outcomes, including transfers, LTFU, and death. While all programs in the cohort had some mechanism for tracing patients lost from care, including notification by home-based caregivers (informal patient-trackers), family members, and residential facilities associated with clinics, it was not standardized across all sites. This may have introduced ascertainment bias, and furthermore, the deaths in the LTFU group are a minimum estimate. Second, detailed information on ART retrieval, and clinic visits that occurred outside of laboratory follow-up were not recorded in the clinical database. Consequently, determinations of LTFU and ILM were based solely upon clinic visits that were tied to laboratory visits, which may have introduced misclassification bias. We were reassured that the predictors of early death (low baseline CD4 count and male sex) and LTFU (male sex) were consistent with those reported in other studies of patient retention and attrition. Finally, use of logistic regression analysis rather than survival analysis did not allow us to calculate incidence of LTFU. Despite these limitations, this study provides a novel assessment of patients lost from HIV care in a large, multi-site, NGO-based clinical program in South Africa, with a focus on the importance of interrupted monitoring and care.

In summary, in the first year of ART initiation in a large multi-site cohort in South Africa, we found that 60% of patients remain in care, 30% miss visits, and 10% die early from advanced immune suppression. Of the patients who miss visits, nearly 40% have interruptions at their primary clinical sites. While most returned to care with virologic suppression in the short term, our study suggests that patients who interrupt care generally, and laboratory monitoring specifically, may have worse virologic outcomes than patients who remain in care. ILM may become more common as ART programs become more widely available, and studies that do not account for such interruptions will overestimate LTFU rates. In the current study, LTFU rates decreased from 2004–2008, while ILM increased. For program evaluation and quality improvement, it is critical to distinguish between these two important outcomes, and specifically to account for patients who are successfully receiving ART in new locations.
